# Evidence for an association between cutaneous malignant melanoma and lymphoid malignancy: a population-based retrospective cohort study in Scotland

**DOI:** 10.1038/sj.bjc.6600692

**Published:** 2003-01-28

**Authors:** D B McKenna, D Stockton, D H Brewster, V R Doherty

**Affiliations:** 1 Department of Dermatology, Royal Infirmary of Edinburgh, Lauriston Place, Edinburgh EH3 9YW, UK; 2 The Scottish Cancer Intelligence Unit, Information and Statistics Division, Trinity Park House, South Trinity Road, Edinburgh EH5 3SQ, Scotland, UK

**Keywords:** epidemiology, malignant melanoma, non-Hodgkin's lymphoma, chronic lymphatic leukaemia

## Abstract

We analysed the risk of cutaneous malignant melanoma (CM) occurring in patients following a diagnosis of non-Hodgkin's lymphoma (NHL) or chronic lymphatic leukaemia (CLL), and of NHL or CLL subsequently developing in CM survivors. Cohorts of patients with CM, NHL or CLL (index cancer) diagnosed between 1975 and 1997 were identified from the Scottish national cancer registry and followed through the registry for subsequent CM, NHL or CLL. The standardised incidence ratio (SIR) for each cancer was calculated and overall risk, risk in relation to gender and age at diagnosis of the index cancers and time from diagnosis of the index cancer to the diagnosis of the second malignancy were measured. There were 9385 CM patients, 4016 CLL patients and 13 857 NHL patients identified with an index cancer with 56 195, 14 450 and 44 999 person-years of follow-up, respectively. There was an increased risk of both CLL and NHL following a diagnosis of CM (SIR 2.3 and 1.5, respectively) and of CM following a diagnosis of CLL and NHL (SIR 2.3 and 2.1, respectively). The risk was statistically significantly increased for CLL developing in CM patients and for CM occurring in NHL survivors (*P*<0.05). This study supports an association between CM, CLL and NHL developing in the same patient. Immunosuppression, exposure to ultraviolet radiation and genetic factors may lead to a host environment that is conducive to the development of these malignancies.

Patients with cutaneous malignant melanoma (CM) are at a significantly increased risk of developing both melanoma (Burden *et al*, 1994) and nonmelanoma skin cancer (Swerdlow *et al*, 1995), which is most likely because of the shared risk factors of exposure to ultraviolet radiation and skin type. The association between CM and other cancer is controversial, but clarification of these relations may lead to an improved understanding of aetiology. Several studies reporting an overall increased incidence of a second primary tumour among CM survivors have been small hospital-based case series or with short follow-up periods or included nonmelanoma skin cancer as a second primary (Adami *et al*, 1995; Swerdlow *et al*, 1995; Wassberg *et al*, 1996; Bhatia *et al*, 1999); the type of secondary tumour reported has been inconsistent (Gutman *et al*, 1991; Riou *et al*, 1995; McKenna *et al*, 2000).

Second cancers including CM have also been well documented among patients with lymphoid malignancy including non-Hodgkin's lymphoma (NHL) and chronic lymphatic leukaemia (CLL) (Travis *et al* 1991,^1992^; Adami *et al*, 1995; Hall *et al*, 1995; Levi *et al*, 1996; Brennan *et al*, 2000). This has been attributed to the cytotoxic and immunosuppressive effects of the drug regimens used to treat these patients (Tucker *et al*, 1988). We have previously described a series of CM patients treated by surgery only, who subsequently developed NHL or CLL, which suggests an association unrelated to chemotherapy (McKenna *et al*, 2000). Using a population-based cancer registry, we examined the possibility of an association between CM and NHL or CLL by calculating the risk of CM occurring in patients following a diagnosis of NHL or CLL, and of NHL or CLL subsequently developing in CM survivors.

## MATERIALS AND METHODS

All patients with a first diagnosis of CM, NHL or CLL (index cancer) between 1975 and 1997 were identified from the Scottish national cancer registration system. Patients with an index cancer were followed through the population-based data set for a subsequent cancer, either CM, NHL or CLL. Only the first cancer occurring after the index cancer was included in the analysis to avoid the potential confounding influence of the second cancer or its treatment on any subsequent malignancy. Person-years at risk were calculated as the difference between the date of diagnosis of the index cancer and either death, date of subsequent cancer or the end of the follow-up period (December 1997), whichever occurred first. Person-years of follow-up were stratified by gender, 5-year age groups, and calendar time periods (1975–1979, 1980–1984, 1985–1989, 1990–1994, 1995–1997). The expected number of cancers for each stratum was calculated by multiplying the person-years at risk in each stratum by the corresponding sex-, age- and calendar period-specific incidence rates for Scotland. These were then added together to obtain the total expected number of cancers. Overall risk and risk in relation to gender and age at diagnosis of the index cancer were analysed. Risk in relation to time since first diagnosis was studied in order to address the influence of medical surveillance and treatment, which would be expected to be greater in the first 2 years of follow-up in the former and greater in the subsequent years for the latter. The standardised incidence ratio (SIR) was defined as the ratio of the observed to expected number of cases. The 95% confidence interval and the test of significance were estimated assuming that the observed number of cases followed a Poisson distribution.

The logarithm of age- and sex-standardised incidence rates, directly standardised to the European standard population, were used to show the changes in incidence rates between 1968 and 1997.

## RESULTS

There were 9385 patients identified with CM as the index cancer with 56 195 person-years of follow-up. There were 3465 males and the median age at diagnosis was 57.3 years. The total number of observed and expected cases of CLL and NHL developing within this group is shown in Tables 1

**Table 1:** SIRs for CLL occurring after a diagnosis of malignant melanoma by sex, age and time from diagnosis of melanoma

**Characteristic**	**Observed**	**Expected**	**SIR**	**95% CI**	***P*-value**
Total	10.0	4.3	2.3	(1.1, 4.4)	<0.05
Male	6.0	2.1	2.9	(1.0, 6.4)	<0.05
Female	4.0	2.2	1.8	(0.5, 4.8)	n.s.
					
*Age at diagnosis*					
<50 years	1.0	0.3	3.8	(0.1, 21.4)	n.s.
≥50 years	9.0	4.0	2.2	(1.0–4.3)	<0.05
					
*Time from CM diagnosis*					
0–2 years	5.0	1.3	4.0	(1.2, 9.3)	<0.05
3–10 years	4.0	2.3	1.7	(0.4, 4.5)	n.s.
≥11 years	1.0	0.7	1.4	(0, 8.2)	n.s.

CM=cutaneous malignant melanoma, SIR=standardised incidence rate, CI=confidence interval, n.s.=not significant.

and 2

**Table 2:** SIRs for NHL occurring after a diagnosis of malignant melanoma by sex, age and time from diagnosis of melanoma

**Characteristic**	**Observed**	**Expected**	**SIR**	**95% CI**	***P*-value**
Total	18.0	12.2	1.5	(0.0, 2.4)	n.s.
Male	9.0	4.4	2.0	(0.9, 3.9)	n.s.
Female	9.0	7.8	1.2	(0.5, 2.2)	n.s.
					
*Age at diagnosis*					
<50 years	4.0	1.8	2.2	(0.6, 5.6)	n.s.
≥50 years	14.0	10.4	1.3	(0.8, 2.4)	n.s.
					
*Time from CM diagnosis*					
0–2 years	10.0	3.6	2.8	(1.3, 5.2)	<0.01
3–10 years	6.0	6.9	0.9	(0.3, 1.9)	n.s.
≥11 years	2.0	1.8	1.1	(0.1, 4.2)	n.s.

See Table 1.

, respectively. These results are also shown for age, sex and time from CM diagnosis. Overall, there was a statistically significant 130% increased risk of CLL recorded following a diagnosis of CM ( Table 1). The risk was greater in males than females, and was significantly raised in the first 2 years following a diagnosis of CM (*P*<0.05), although it remained elevated throughout the follow-up period. The risk was increased, regardless of age, but this was only statistically significant for patients aged more than 50 years at diagnosis of CM (*P*<0.05), there being very few cases of CM diagnosed under the age of 50.

There was a nonsignificant 50% higher risk of developing NHL following a diagnosis of CM which was higher in males than females ( Table 2). The increased risk of NHL following CM was restricted to the first 2 years of follow-up (*P*<0.01). The risk of NHL was greater in patients with a diagnosis of CM before the age of 50 years, but this was not statistically significant.

There were 4016 patients identified with CLL as the index cancer with 14 450 person-years of follow-up. There were 2351 males and the median age at diagnosis was 72.2 years. The total number of observed and expected cases of CM developing within this group is shown in Table 3

**Table 3:** SIRs for malignant melanoma occurring after a diagnosis of CLL by sex, age and time from diagnosis of CLL

**Characteristic**	**Observed**	**Expected**	**SIR**	**95% CI**	***P*-value**
Total	6.0	2.6	2.3	(0.0, 2.4)	n.s.
Male	4.0	1.4	2.9	(0.9, 3.9)	n.s.
Female	2.0	1.6	1.6	(0.5, 2.2)	n.s.
					
*Age at diagnosis*					
<50 years	0.0	0.1	0.0	(0.0, 0.0)	n.s.
≥50 years	6.0	2.5	2.4	(0.9, 5.2)	n.s.
					
*Time from CLL diagnosis*					
0–2 years	4.0	1.0	3.9	(1.0, 10.0)	<0.05
3–10 years	2.0	1.4	1.4	(0.1, 5.3)	n.s.
≥11 years	0.0	0.2	0.0	(0.0, 0.0)	n.s.

CLL=chronic lymphatic leukaemia, SIR=standardised incidence rate, CI=confidence interval, n.s.=not significant.

, also stratified by age, sex and time from CLL diagnosis. Overall, there was a nonsignificant 130% increased risk of CM developing following a diagnosis of CLL which was greater in males than females. The risk of detecting CM was significantly increased within the first 2 years of a diagnosis of CLL (*P*<0.05), and was still greater than expected 3–10 years later. Only those patients with a diagnosis of CLL after the age of 50 years had an elevated risk of developing CM.

There were 13 857 patients identified with NHL as the index cancer with 44 999 person-years of follow-up. There were 6760 males and the median age at diagnosis was 67.0 years. The total number of observed and expected cases of CM developing within this group is shown in Table 4

**Table 4:** SIRs for malignant melanoma occurring after a diagnosis of NHL by sex, age and time from diagnosis of NHL

**Characteristic**	**Observed**	**Expected**	**SIR**	**95% CI**	***P*-value**
Total	14.0	6.6	2.1	(1.2, 3.6)	<0.05
Male	10.0	2.6	3.8	(1.8, 7.0)	<0.001
Female	4.0	4.0	1.0	(0.3, 2.6)	n.s.
					
*Age at diagnosis*					
<50 years	2.0	1.1	1.8	(0.2, 6.4)	n.s.
≥50 years	12.0	5.5	2.2	(1.1, 3.9)	<0.05
					
*Time from NHL diagnosis*					
0–2 years	6.0	2.6	2.3	(0.8, 5.1)	n.s.
3–10 years	7.0	3.4	2.1	(0.8, 4.3)	n.s.
≥11 years	1.0	0.7	1.5	(0.0, 8.8)	n.s.

NHL=non-Hodgkin's lymphoma, SIR=standardised incidence rate, CI=confidence interval, n.s.=not significant.

, also stratified by age, sex and time from CM diagnosis. Overall, there was a statistically significant 110% increased risk of CM developing in patients with a previous diagnosis of NHL (*P*<0.05), although this excess was only observed in males (*P*<0.001). The risk of CM developing within the first 2 years of NHL diagnosis was elevated and remained higher than expected throughout the period of follow-up, although not statistically significant. The risk was higher and statistically significant in patients who had been diagnosed with NHL aged 50 years or older.

## DISCUSSION

**Figure 1:**
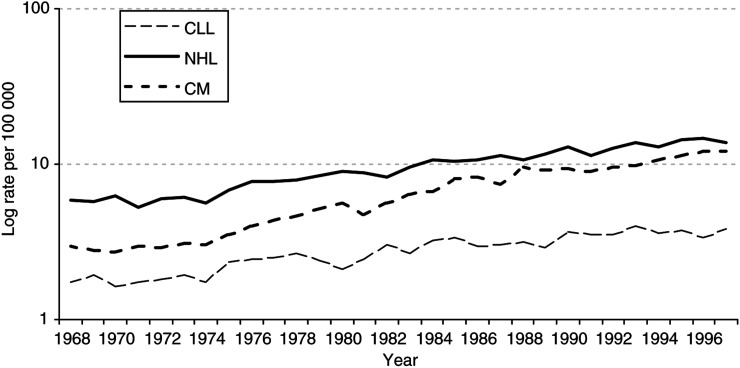
Age- and sex-standardised incidence rates for selected cancers: 1968–1997 European age-standardised rates per 100 000 population. CM=cutaneous malignant melanoma, CLL=chronic lymphatic leukaemia, NHL=non-Hodgkin's lymphoma.

The rising incidence of both CM and of NHL and CLL over the last two decades has led some investigators to suggest a common aetiological association (Figure 1

). The rate of increase has been greatest for CM, which has more than trebled in the last 20 years. This rise has been attributed to increasing levels of exposure to ultraviolet radiation although the dosage and pattern of exposure implicated in its development are unclear (Gilchrest *et al*, 1999). Apart from the acquired immunodeficiency syndrome and an expanding population of transplant recipients on immunosuppressive therapy, the increasing incidence of lymphoproliferative disorders has remained unexplained (Bray *et al*, 2001). In this study, we found an increased risk of both NHL and CLL following a diagnosis of CM and *vice versa* which was statistically significant for CLL following a diagnosis of CM and for CM in NHL survivors.

There are several theoretical explanations for the development of CM and CLL or NHL in the same patient. Chronic immunosuppression is an established risk factor for the development of both lymphoid malignancy and skin cancer. An increase in CM has been reported following Hodgkin's (Tucker *et al*, 1988) and non-Hodgkin's lymphoma (Levi *et al*, 1996), CLL (Travis *et al*, 1992), renal transplantation (Greene *et al*, 1981), immunosuppressive therapy (Kinlen, 1985) and among those with genetically determined immunodeficiency diseases (Spector *et al*, 1978). CM that develop in immunosuppressed patients tends to originate from dysplastic naevi, are more deeply invasive with marked depression of a host lymphocyte–macrophage response and have a poorer prognosis (Tucker *et al*, 1988). Acquired or iatrogenic immunosuppression may impair cell-mediated immunity such that tumour-specific lymphocytes are unable to maintain an effective tumour surveillance system. The cytotoxic and immunosuppressive effects of the chemotherapeutic regimens used in the treatment of NHL patients could explain the higher risk for CM in NHL survivors as in our study. The risk of CM in CLL patients and *vice versa* was identical, consistent with the potentially indolent course of CLL, which usually presents in elderly patients and often requires no treatment (Rozman *et al*, 1997). Moreover, patients with NHL and CLL exhibit a variety of immunological defects that may also increase their risk of CM in the absence of any treatment effect (Vanhaelen and Fisher, 1982).

The power to detect a statistically significant excess risk of a second cancer in patients whose index malignancy was diagnosed before the age of 50 years was limited by relatively small numbers of index cases below this age cutoff. Nevertheless, the higher risk of CM in patients with a diagnosis of NHL or CLL after the age of 50 years may be a result of cumulative exposure to ultraviolet radiation followed by immunosuppression as a consequence of NHL, CLL or its treatment. The much higher risk of CM in male NHL and CLL patients is noteworthy and has been recorded in other studies (Travis *et al*, 1991; 1992; Tsao *et al*, 2002). We have no evidence that males are more aggressively treated than females or are more susceptible to developing skin cancer as a consequence of the treatment used for these patients.

The relative risk of CM was highest in the first 2 years following the diagnosis of either NHL or CLL, and remained elevated throughout in NHL patients. The higher SIR early on after NHL or CLL diagnosis suggests a possible detection bias because of increased medical surveillance of patients with malignancy. Although this may have resulted in a small increase in the overall SIR, the main effect of this bias would be to bring forward in time the diagnosis of some cases of CM that may have gone undetected until several years later.

Following a diagnosis of CM, there was a significant 2.3-fold increased risk of CLL, which was greatest in the first 2 years following the CM and was higher in males than females ( Table 1). Similarly, there was a 50% increased risk of NHL in CM survivors, which was significant in the first two years of follow-up and which was also higher in males ( Table 2). The significantly higher risk of CLL developing after the age of 50 years may be in keeping with the average age of onset of the CLL of 70 years (Rozman *et al*, 1997). These results are consistent with the findings of other population-based cancer registry studies, which have shown an increased risk of 1.4–2.0 for NHL and CLL following CM (Adami *et al*, 1995; Hall *et al*, 1995; Wassberg *et al*, 1996; Goggins *et al*, 2001). Although we do not have any information on treatment, it is unlikely that either radiotherapy or chemotherapy contributed to the development of CLL or NHL in these patients as surgery is the main therapeutic intervention for CM. Whether these patients have a host environment conducive to the development of both CM and lymphoid malignancy remains to be determined. Several tumour-related immunodeficiency mechanisms, mediated by the neoplasm itself, have been identified and appear to be responsible for inhibiting recognition and destruction of tumour cells in these patients. Persistent suppressor T-cell activity has been described in long-term survivors with lymphoma (Vanhaelen and Fisher, 1982) and enhanced suppressor T-cell activity has been demonstrated in patients with CM, which disappears with removal of the tumour (Werkmeister *et al*, 1981). Such a mechanism may explain an aetiological link between melanoma and the occurrence of lymphoid malignancy in the same patient.

In addition to its mutagenic effect, ultraviolet radiation (UV) has an immunosuppressive effect both systemically and locally in the skin (Cooper *et al*, 1992). Several epidemiological studies have linked the parallel rise in incidence of lymphoid disorders and skin cancer to increased levels of UV exposure (Adami *et al*, 1995,^1999^; McMichael and Giles, 1996), although this has not been confirmed by others (Hartge *et al*, 1996; Freedman *et al*, 1997). An excess risk of both CM and lymphoid malignancy has been reported in higher socioeconomic, white-collar workers (Schumacher and Delzell, 1988; MacKie and Hole, 1996), possibly because of increased recreational sun exposure. In a geographically based study in England and Wales, the incidence of NHL was significantly associated with high levels of UV exposure, even after correction for social class and occupation (Bentham, 1996). Other studies using skin cancer as a marker of UV exposure have shown a positive correlation between lymphoid disorders and skin cancer, including CM (Adami *et al*, 1995; Hall *et al*, 1995; Tsao *et al*, 2002).

Both CM and lymphoid malignancy have a strong genetic component. The p16 gene, which inhibits the cyclin-dependent kinase CDNK4, has been proposed as a candidate for a tumour suppressor gene located on chromosome 9p21 (Kamb *et al*, 1994). Mutations and deletions of this gene have been reported in a wide variety of human cancers, including familial and sporadic cases of CM (Healy *et al*, 1996) as well in several different types of leukaemia and lymphoma (Ogawa *et al*, 1994). Molecular screening of tissue samples for inactivation of this gene in patients who develop both CM and lymphoid malignancy may determine whether it is important in the common pathogenesis of these tumours.

In summary, we found an increased risk of both NHL and CLL following a diagnosis of CM and *vice versa* that suggests an association. In addition to treatment effects and surveillance bias, further study is required to determine what role, if any, shared environmental or genetic factors play in this association.
